# Supplementary material for: Determining spin-orbit coupling in graphene by quasiparticle interference imaging
Source: Nat Commun. 2023 Jun 24;14:3771. doi: 10.1038/s41467-023-39453-x (PMC10290717; doi:10.1038/s41467-023-39453-x)
Supplement: Supplementary file 1 — Supplementary Information [file 41467_2023_39453_MOESM1_ESM.pdf]

Supplementary Material: Determining spin-orbit coupling in graphene by quasiparticle interference spectroscopy

Lihuan Sun,[1] Louk Rademaker,[1,2] Diego Mauro,[1,3] Alessandro Scarfato,[1] Árpád Pásztor,[1] Ignacio Gutiérrez-Lezama,[1,3] Zhe Wang,[4,1] Jose Martinez-Castro,[1] Alberto F. Morpurgo,[1,3] and Christoph Renner[1]

[1]*Department of Quantum Matter Physics, University of Geneva, 1211 Geneva, Switzerland*

[2]*Department of Theoretical Physics, University of Geneva, 1211 Geneva, Switzerland*

[3]*Group of Applied Physics, University of Geneva, 1211 Geneva, Switzerland*

[4]*MOE Key Laboratory for Nonequilibrium Synthesis and Modulation of Condensed Matter, Shaanxi Province Key Laboratory of Advanced Materials and Mesoscopic Physics, School of Physics, Xi'an Jiaotong University, 710049, Xi'an, China*

(Dated: June 5, 2023)

S1. CONFIRMING THE TWIST ANGLE OF THE MEASURED DEVICES

We prepared SLG-on-WSe_2 heterostructures with nominal twist angles of 1° to 30° between the exfoliated SLG and the WSe_2 lattices. The actual twist angle of each measured device is determined before the QPI experiments based on high resolution topographic STM images displayed in Supplementary Figure S1.

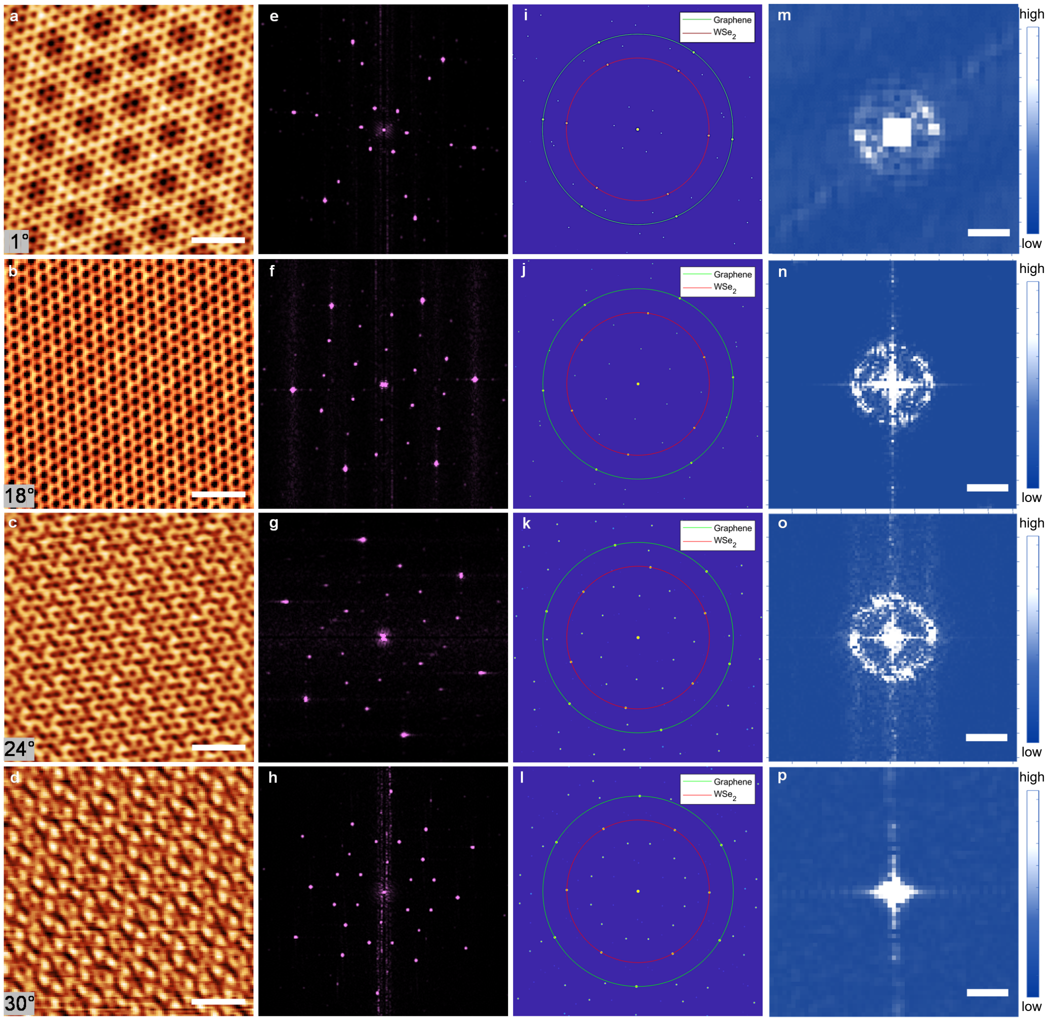

Figure S1. **Twist angle dependent topography and QPI of SLG-on-WSe_2 heterostructures.** **a-d**, Topographic STM images of 1°, 18°, 24° and 30° twist angle heterostructures (scale bar, 1 nm). **e-h**, Fourier transform of the experimental topographic data in **a-d**. **i-l**, corresponding model calculation FTs based on the method described in [1]. **m-p**, typical QPI maps around the Γ-point for each twist angle (scale bar, 0.5 nm^{-1}).

S2. THEORETICAL CALCULATION

A. Weak coupling theory of QPI

The outcome of an STM measurement is the *local tunneling density of states* $N(\mathbf{x}, \omega) = \frac{dI(V,\mathbf{x})}{dV}$ depending on position $\mathbf{x}$, and where $\omega = eV$ is the frequency associated with a bias voltage V. Here $I(V, \mathbf{x})$ is the tunneling current as a function of voltage V and position $\mathbf{x}$. The local density of states is related to the *single-particle Greens function* $G(\mathbf{x}, \mathbf{x}'; \omega)$ as follows,

$$N(\mathbf{x}, \omega) = -\frac{1}{\pi}\mathrm{Im}\left[G(\mathbf{x}, \mathbf{x}; \omega)\right]. \tag{S1}$$

The QPI spectrum is the Fourier transform of the local density of states,

$$P(q, \omega) = \left|\frac{1}{N}\sum_{x} e^{i\mathbf{k}\mathbf{x}} N(\mathbf{x}, \omega)\right|. \tag{S2}$$

When the disorder potential is weak, you can construct an approximate solution of the QPI spectrum known as *weak coupling theory.* In first order in the disorder potential $V_{\mathbf{k}\mathbf{k}'}$, the change in the Greens function due to disorder is

$$\delta G(\mathbf{k}, \mathbf{k}'; \omega) = G_0(\mathbf{k}; \omega) V_{\mathbf{k}\mathbf{k}'} G_0(\mathbf{k}'; \omega) \tag{S3}$$

where $G_0(\mathbf{k}, \omega)$ is the disorder-free Greens function and $V_{\mathbf{k}\mathbf{k}'}$ is the disorder potential. The power spectrum then becomes

$$P(\mathbf{q}, \omega) = \left|\frac{1}{\pi}\mathrm{Im}\Lambda(\mathbf{q}; \omega)\right| |V(\mathbf{q})| \tag{S4}$$

where $V(\mathbf{q})$ is the Fourier transform of the disorder potential, and we define the Λ function as

$$\Lambda(\mathbf{q}; \omega) \equiv \frac{1}{N_k}\sum_{\mathbf{k}} G_0(\mathbf{k} + \mathbf{q}; \omega) G_0(\mathbf{k}; \omega). \tag{S5}$$

In the remainder of our calculation, we do not take into account any momentum-depedence of the scattering potential $V(\mathbf{q})$. Note that a completely local (Dirac delta function) impurity potential would result in a momentum-independent $V(\mathbf{q})$. More realistic impurity potentials fall off at large $\mathbf{q}$, but this effect is negligible at the small momenta relevant for intravalley scattering we are looking at.

B. Intravalley QPI in graphene without SOC

The low-energy Hamiltonian of graphene without spin-orbit coupling is

$$\hat{H}_0^{(\tau)}(\mathbf{k}) = -v(\tau^z\sigma^x k_x + \sigma^y k_y) + m\sigma^z \tag{S6}$$

where v_F is the Dirac velocity, $\tau^z = \pm 1$ the K/K' valley index, and σ are Pauli matrices representing the sublattice pseudospin, and we included a possible mass term m. The corresponding non-interacting disorderfree Matsubara Greens function is

$$\hat{G}^{(\tau)}(\mathbf{k}; i\omega_n) = \frac{i\omega_n \hat{1} + \hat{H}_0^{(\tau)}(\mathbf{k})}{\omega_n^2 + v_F^2 k^2 + m^2} \tag{S7}$$

A generic disorder potential can act different on the two sublattices. In the weak-coupling approach, we can separately calculate the response from a potential that acts the same on both sublattices (σ^0) and the response from a potential that acts oppositely on the sublattices (σ^3). For both cases, the intravalley Λ function reads

$$\Lambda_\alpha^{(\tau)}(\mathbf{q}; i\omega_n) = \int \frac{d^2k}{(2\pi)^2} \mathrm{Tr}\left[\hat{G}^{(\tau)}(\mathbf{k}; i\omega_n)\sigma^\alpha \hat{G}^{(\tau)}(\mathbf{k}+\mathbf{q}; i\omega_n)\right]. \tag{S8}$$

Using analytic continuation $i\omega_n \to \omega + i\delta$ and the identity $\mathrm{Im}\frac{1}{x+i\delta} = -\pi\delta(x)$ we find exact solutions for Λ_0,

$$\mathrm{Im}\,\Lambda_0(q,\omega) = \frac{1}{v_F^2}\left(1 - \Theta[vq - 2\sqrt{\omega^2 - m^2}]\frac{(v_F q)^2 - (2\omega)^2}{v_F q\sqrt{(v_F q)^2 - (2\omega)^2 + (2m)^2}}\right) \tag{S9}$$

and for Λ_3,

$$\mathrm{Im}\,\Lambda_3(q,\omega) = \Theta[v_F q - 2\sqrt{\omega^2 - m^2}]\frac{4m^2\omega}{v_F^3 q\sqrt{(v_F q)^2 - (2\omega)^2 + (2m)^2}}. \tag{S10}$$

In the absence of a mass term, there is no backscattering peak at $q_B = 2\sqrt{\omega^2 - m^2}/v_F$. However, when $m > 0$, there is a sharp peak at q_B and a $1/q^2$ tail for larger momenta.

C. Intravalley QPI in graphene with SOC

The Hamiltonians for valley-Zeeman (vZ) and Rashba (R) spin-orbit coupling are

$$\hat{H}_{\mathrm{vZ}} = \lambda_{\mathrm{vZ}}\tau^z s^z, \tag{S11}$$

$$\hat{H}_{\mathrm{R}} = \lambda_{\mathrm{R}}(\tau^z\sigma^x s^y - \sigma^y s^x) \tag{S12}$$

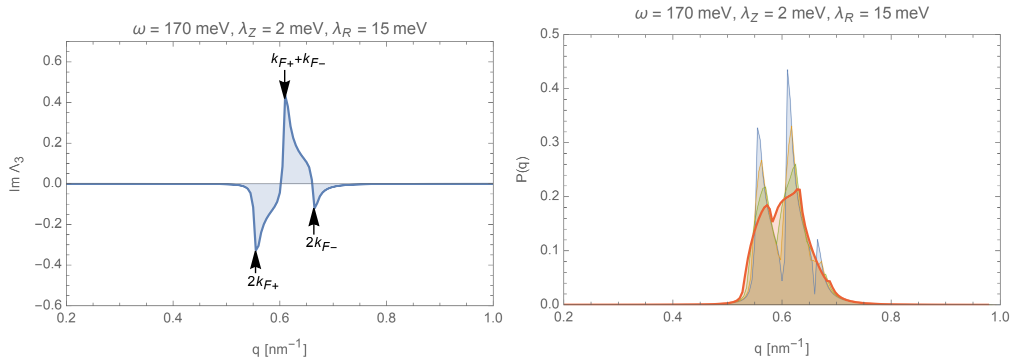

Figure S2. **Left:** The weak coupling QPI spectrum in case of a σ^3 disorder potential in the presence of SOC. On the left we show the exact result, following Eq. (S18). There is a large peak at $q_B = 2k^*_\pm$ for each of the spin-split bands. When two *different* bands are connected, the resulting scattering has the opposite sign. As a result, there are three distinct peaks in the QPI spectrum. **Right:** When experiments have a limited field of view, the momentum resolution is limited as well. We can smear $|\text{Im}\Lambda|$ by a moving average, to obtain the spectrum that can be compared with experiments. Shown are three different degrees of momentum-smearing.

where s are Pauli matrices referring to the spin of the electron. With inclusion of these terms, the spin-split bands become

$$\epsilon_{\mathbf{k}\pm} = \sqrt{(v_F k)^2 + 2\lambda_{\rm R}^2 + \lambda_{\rm vZ}^2 \pm \sqrt{(\lambda_{\rm R}^2 + \lambda_{\rm vZ}^2)(v_F k)^2 + \lambda_{\rm R}^4}}. \tag{S13}$$

Because of the spin splitting, there are now *two* momenta $k^*_\pm$ where the bands $\epsilon_{\mathbf{k}\pm}$ cross a fixed energy ω,

$$(v_F k^*_\pm)^2 = \lambda_{\rm vZ}^2 + \omega^2 \mp 2\sqrt{(\lambda_{\rm vZ}^2 + \lambda_{\rm R}^2)\omega^2 - \lambda_{\rm R}^2\lambda_{\rm vZ}^2}. \tag{S14}$$

The calculation of Λ is quite tedious and lengthy, but can be done analytically. Since the resulting equation is very long, we will describe here the relevant steps for the case of Λ_3. Explicitly, this becomes

$$\Lambda_3(\mathbf{q}; i\omega_n) = 64\lambda_{\rm R}^2\lambda_{\rm vZ} i\omega_n \int \frac{d^2k}{(2\pi)^2} \frac{(v_F k)^2 + v_F^2 \mathbf{k}\cdot\mathbf{q} + 2\lambda_{\rm R}^2 + \lambda_{\rm vZ}^2 + \omega_n^2}{A(\mathbf{k}, i\omega_n)A(\mathbf{k}+\mathbf{q}, i\omega_n)} \tag{S15}$$

$$\equiv \int \frac{d^2k}{(2\pi)^2} \frac{f_3(\mathbf{k}, \mathbf{q}, i\omega_n)}{A(\mathbf{k}, i\omega_n)A(\mathbf{k}+\mathbf{q}, i\omega_n)} \tag{S16}$$

where we have implicitly defined f_3 and the denominator term is

$$\begin{aligned} A(\mathbf{k}, i\omega_n) &= \left[(v_F k)^4 - 2(v_F k)^2\lambda_{\rm vZ}^2 + \lambda_{\rm vZ}^2(\lambda_{\rm vZ}^2 + 4\lambda_{\rm R}^2)\right] + \left[2\lambda_{\rm vZ}^2 + 4\lambda_{\rm R}^2 + 2(v_F k)^2\right]\omega_n^2 + \omega_n^4 \\ &= (\omega_n^2 + \epsilon_{\mathbf{k}+}^2)(\omega_n^2 + \epsilon_{\mathbf{k}-}^2). \end{aligned} \tag{S17}$$

Note that this shows that Λ_3 is only nonzero when *both* valley-Zeeman *and* Rashba SOC are nonzero, which explains why there is no backscattering observed in the 30° device where the valley-Zeeman vanishes by symmetry. The imaginary part of Λ_3, that enters in the expression for the QPI, is now given by

$$\mathrm{Im}\,\Lambda_3(\mathbf{q},\omega) = \sum_{\pm} \frac{\mp 1}{\sqrt{\lambda_{\mathrm{R}}^4 + (v_F k_{\pm}^*)^2(\lambda_{\mathrm{R}}^2 + \lambda_{\mathrm{vZ}}^2)} \pm (\lambda_{\mathrm{R}}^2 + \lambda_{\mathrm{vZ}}^2)} \int \frac{d\theta}{32\pi v_F^2} \left[\frac{f_3(\mathbf{k}_{\pm}^*, \mathbf{q})}{A(\mathbf{k}_{\pm}^* + \mathbf{q})} + \frac{f_3(\mathbf{k}_{\pm}^* - \mathbf{q}, \mathbf{q})}{A(\mathbf{k}_{\pm}^* - \mathbf{q})} \right] \tag{S18}$$

where the dependence on the energy ω is contained within the Fermi momentum k_F, and the remaining integrals over the angle θ between $\mathbf{k}$ and $\mathbf{q}$ are of the form

$$\int_0^{2\pi} \frac{d\theta}{2\pi} \frac{1}{a + b\cos\theta} = \frac{\Theta[a^2 - b^2]}{\sqrt{a^2 - b^2}}, \tag{S19}$$

$$\int_0^{2\pi} \frac{d\theta}{2\pi} \frac{\cos\theta}{a + b\cos\theta} = \frac{1}{b} - \frac{a}{b} \frac{\Theta[a^2 - b^2]}{\sqrt{a^2 - b^2}}. \tag{S20}$$

Even though the result is analytically exact, writing down the equation takes several pages and we therefore do not put it here. We used Mathematica to express the final result. A typical figure of the resulting QPI spectrum is shown in Fig. S2. We see three peaks, associated with $q_B = 2k_+^*$, $2k_-^*$ and $k_+^* + k_-^*$. Note that for larger momenta, there is no appreciable QPI scattering.

A similar calculation for Λ_0 was performed.

D. Comparison to experiment

In order to compare our exact theoretical results to the experiments, we performed a moving average of the theoretical power spectrum $P(q)$ to account for the limited momentum resolution. The smearing parameter for moving average is taken as $0.1\,\mathrm{nm}^{-1}$, which is similar to momentum resolution($0.03\,\mathrm{nm}^{-1}$) in experiments. The momentum resolution we consider here is determined by dI/dV map size in experiments.

Additionally, since we are unaware of the precise disorder potential shape, we took a sum of the Λ_0 and Λ_3 power spectra to fit the experimental results. The proportion weight of Λ_0 and Λ_3 are set as 0.01 and 0.99 in the fitting. Furthermore, note that typically the disorder potential in momentum space falls off with increasing momentum. As a result, the *intravalley* scattering due to SOC are stronger visible in the QPI than the large-momentum *intervalley* scattering, which is consistent with our experimental results.

The valley-Zeeman and Rashba SOC are extracted by adjusting the theoretical model to the QPI profile in momentum space. The calculated QPI depends on the following parameters: the valley-Zeeman and the Rashba SOC terms, the sample bias, the Fermi velocity, and the finite momentum resolution of the experimental data given to first order by the image size. Since there are two different kinds of disorder, we use the sum of Λ_0 and Λ_3 power spectra to fit the experimental results. The free parameters are the valley-Zeeman and the Rashba SOC amplitudes. The most suitable valley-Zeeman and Rashba terms are obtained by adjusting the calculated backscattering amplitude to the experimental QPI profile. The width of the backscattering ring is primarily given by the Rashba term and its amplitude by the vZ term.

S3. ANGULAR AVERAGING OF THE QPI TRACES IN MOMENTUM SPACE

In Supplementary Figure S3, we show angle-averaged QPI traces in momentum space to demonstrate that the peak at q_B does not change significantly when integrating the signal over increasingly large angles between 5 and 90 degrees.

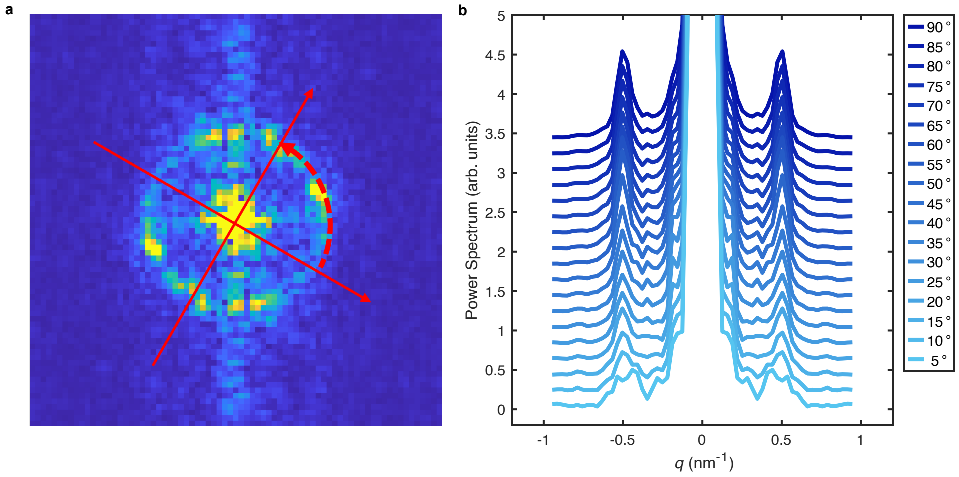

Figure S3. **Angular averaging of QPI traces.** **a**, FT of the QPI image of a 24° twist angle device, same as Fig. 5a in the main text. **b**, QPI traces averaged over increasingly large angles in momentum space indicated by the red arrow from 0° to 90° in **a**.

S4. STM TOPOGRAPHY AND CONDUCTANCE MAP OF A 30° TWIST ANGLE HETEROSTRUCTURE

In Supplementary Figure S4, we present a complete data set with STM topography, QPI imaging and tunneling spectroscopy of a 30° twist angle SLG-on-WSe_2 heterostructure. The $dI/dV(V)$ spectroscopy in Fig. S4d indicates a Dirac point near V=-145 meV. The $dI/dV(V = 50mV, \vec{r})$ map in Fig. S4f measured over the area imaged in Figure S4e (the same topography is presented in the main text) clearly shows that there are no long wavelength periodic charge modulations which would be expected in the presence of backscattering. The central region near the Γ point of the FT of Figure S4f, presented in in Fig. 5f of the main text, shows there is no intensity at the expected backscattering momenta.

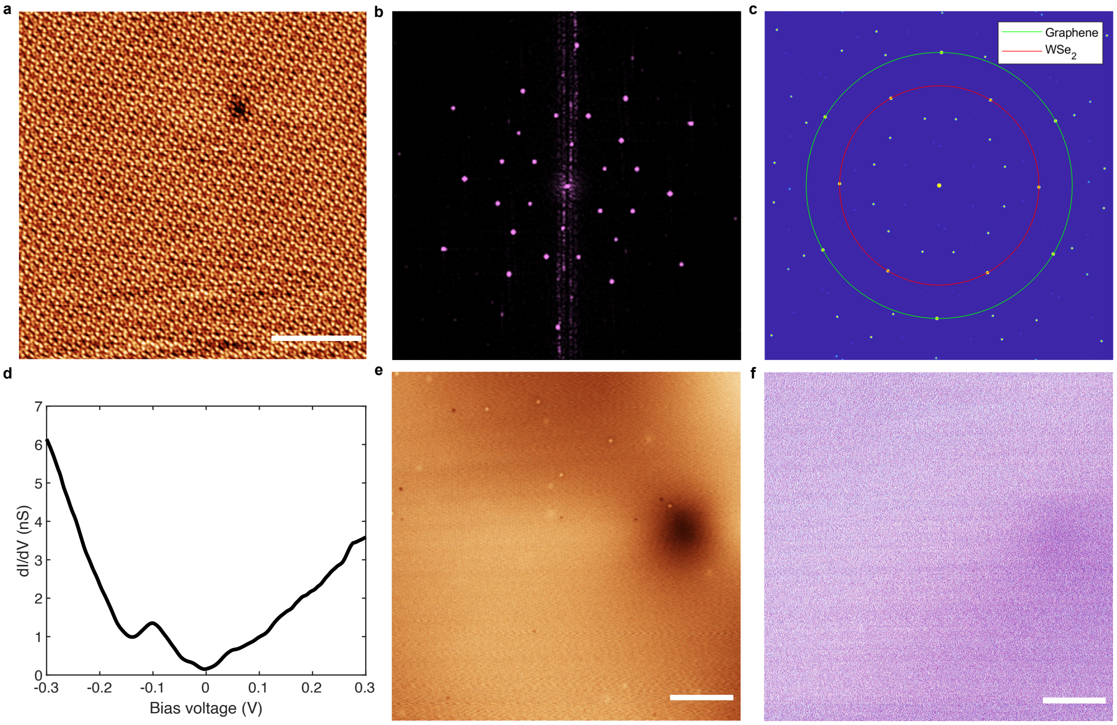

Figure S4. **Topographic and spectroscopic STM characterization of a SLG-on-WSe_2 heterostructure with a twist angle of 30°.** **a**, STM topographic image (scale bar, 5 nm) and **b**, corresponding FT. **c**, Model calculation of the FT expected for a 30° SLG/WSe_2 heterostructure. **d**, Typical $dI/dV(V)$ spectrum showing the phonon gap at the Fermi level ($V = 0$ V) and the Dirac point near $V =$ -145 meV. **e**, Topographic STM image over a larger area with defects, and **f**, corresponding $dI/dV(V = 50\,mV, \vec{r})$ map (scale bars, 20 nm).

[1] Martinez-Castro, J. *et al.* Scanning tunneling microscopy of an air sensitive dichalcogenide through an encapsulating layer. *Nano Lett* **18**, 6696–6702 (2018).
